# Genome-Wide Association Study for Milk Protein Content in Romanian Dual-Purpose Cattle

**DOI:** 10.3390/life15111668

**Published:** 2025-10-26

**Authors:** Daniel George Bratu, Șerban Blaga, Bianca Cornelia Zanfira, Călin Mircu, Ioana Irina Spătaru, Iuliu Torda, Alexandru Eugeniu Mizeranschi, Daniela Elena Ilie, Ludovic Toma Cziszter, Dorin Alexandru Vizitiu, Oana Maria Boldura, Ioan Huțu

**Affiliations:** 1 Horia Cernescu Research Unit, Faculty of Veterinary Medicine Timisoara, University of Life Science “Regele Mihai I” from Timisoara, Calea Aradului 119, 300645 Timisoara, Romania; daniel.bratu@usvt.ro (D.G.B.); lungu.bianca@usvt.ro (B.C.Z.); calinmircu@usvt.ro (C.M.); irina.spataru@usvt.ro (I.I.S.); iuliu.torda@usvt.ro (I.T.); dorin.vizitiu@usvt.ro (D.A.V.); oanaboldura@usvt.ro (O.M.B.); ioan.hutu@fmvt.ro (I.H.); 2 Extension Unit and Advisory Center, 300645 Timisoara, Romania; cziszterl@animalsci-tm.ro; 3 Research and Development Station for Bovine Arad, 310059 Arad, Romania; alexandru.mizeranschi@scdcbarad.ro; 4 Institute for Advanced Environmental Research, West University of Timisoara, 300223 Timisoara, Romania; 5 Faculty of Bioengineering of Animal Resources, University of Life Sciences “Regele Mihai I” from Timisoara, Calea Aradului 119, 300645 Timisoara, Romania

**Keywords:** Romanian dual-purpose cattle, milk protein content, genome-wide association study, quantitative trait loci

## Abstract

Milk protein content represents a key economic trait in dairy production, yet the genetic architecture underlying this trait remains unexplored in Romanian dual-purpose cattle breeds. This study conducted a genome-wide association analysis for milk protein content in 313 Romanian Simmental (n = 271) and Romanian Brown (n = 42) cows belonging to the Research and Development Station for Bovine Arad, Romania. Following quality control, 33,531 SNPs were tested for association with protein percentage adjusted for other effects (breed, days in milk, season, year, parity) using linear regression with the first five principal components as covariates to control population stratification. Although no SNP reached genome-wide significance (*p* < 5 × 10^−8^), one SNP achieved significance (*p* < 2.98 × 10^−5^) and seven additional SNPs exceeded the nominal threshold (*p* < 1 × 10^−4^) across six chromosomes. The strongest association (*p* = 9.56 × 10^−6^) mapped to chromosome 25 near *C7orf61*. Biologically relevant candidate genes included *KLF6* on chromosome 13, previously associated with milk traits in Chinese Holstein, and *AHCYL1* on chromosome 3, involved in calcium homeostasis. These findings provide initial insights into genomic regions influencing milk protein content in Romanian dual-purpose cattle, though validation in larger cohorts needs to be carried out.

## 1. Introduction

Milk protein content represents a critical economic trait in dairy cattle production, directly influencing cheese yield and milk payment schemes worldwide. In New Zealand’s dairy industry, each additional kilogram of protein generates approximately $7.10 NZD in annual profit [1], while European markets similarly prioritize protein content in milk pricing schemes. Romanian animal breeding programs also prioritize economically significant traits, including milk protein content [2]. The economic significance of this trait has driven extensive genetic selection programs globally, making it a priority to identify genomic regions that control milk protein synthesis.

Milk protein content demonstrates moderate to high heritability across cattle populations, varying substantially by breed and lactation stage. In Holstein populations, heritability estimates for milk protein percentage range from 0.07 to 0.16, while protein yield shows values from 0.14 to 0.27 [3]. Danish Jersey cattle demonstrate considerably higher heritability values, reaching 0.70 for protein percentage [4]. Dutch Holstein-Friesians show heritability values ranging from 0.25 for β-casein to 0.80 for β-lactoglobulin concentrations [5]. These substantial genetic components indicate significant potential for genomic selection to improve protein content in dairy herds.

Genome-wide association studies (GWAS) have successfully identified numerous quantitative trait loci (QTL) influencing milk protein composition across major dairy breeds. A comprehensive meta-analysis of 94,321 cattle from eight breeds identified 176 QTL for protein percentage, with 114 showing consistent effects across breeds [6]. The most significant associations consistently map specific chromosomal regions: BTA6 harboring the casein gene cluster (*CSN1S1, CSN1S2, CSN2, CSN3*) [7], *BTA14* containing *DGAT1* and *BTA20* containing growth hormone receptor genes [8], and *BTA11* with the *PAEP* gene encoding β-lactoglobulin [6].

Romanian dual-purpose cattle represent unique genetic resources adapted to local environmental conditions, yet remain largely unexplored through genomic approaches. Genome-wide association studies (GWAS) have been conducted on the Romanian Simmental and Brown breeds [9], alongside the Romanian Grey Steppe [10]. The Romanian Grey Steppe is a direct descendant of *Bos taurus primigenius*, and it now has numbers fewer than 100 animals nationwide, representing a critically endangered breed [11].

Romanian Simmental and Romanian Brown represent two economically significant dual-purpose cattle breeds in Romania. Romanian Simmental was developed through long-term, systematic crossbreeding between local Romanian Grey cattle and Simmental-type bulls imported from multiple countries, including Austria, Germany, Switzerland, the Czech Republic, and Slovakia. Romanian Brown was established through crosses between Schwyz bulls and Grey Steppe or Mountain Breed cows [12,13].

Both breeds demonstrate substantial phenotypic variation in milk production traits suitable for genomic association studies. 

Romanian Simmental cows produce an average of 5419 ± 113 kg of milk per 305-day lactation, with a recorded daily average of 18.10 kg of milk containing 3.96% fat and 3.46% protein [2]. The protein content varies according to genetic markers at the CSN3 locus: cows with the CSN3 AA genotype produce 3.27 ± 0.03% protein, whereas those with the BB genotype yield 3.40 ± 0.02% (*p* ≤ 0.05) [14], indicating significant genetic variation. Romanian Brown demonstrates average milk yields of 4897 ± 129 kg per 305-day lactation with protein content of 3.24 ± 0.03% [12], though prolactin gene polymorphisms reveal protein percentages reaching 3.96 ± 0.32% in favorable AA genotypes compared to 3.43 ± 0.15% in GG genotypes (*p* = 0.027) [15]. Despite their distinct genetic origins (Romanian Simmental deriving from Central European Simmental lineages and Romanian Brown from Swiss Alpine cattle), both breeds demonstrate exceptional genetic diversity [2]. Microsatellite analysis reveals observed heterozygosity of 0.895 for Romanian Simmental and 0.966 for Romanian Brown [10].

The genetic architecture of complex traits like milk protein content involves numerous genes with small effects, requiring adequate sample sizes and appropriate statistical models to detect associations. Recent cattle GWAS have employed increasingly large cohorts, with the largest Holstein study analyzing 294,079 animals [8]. However, for populations with limited census, like Romanian breeds, alternative approaches maximizing statistical power while controlling for population structure become essential. The genomic inflation factor (*λ*) serves as a measure of systematic bias in association statistics, with values above 1.2 classified as large and substantially affecting statistical power in GWAS meta-analyses [16].

Understanding the genetic architecture of milk protein synthesis in Romanian Simmental and Romanian Brown cattle could identify breed-specific variants, contribute to conservation efforts, and enhance genomic selection programs for local dairy production. Therefore, this study aimed to conduct the first GWAS for milk protein content in Romanian dual-purpose cattle breeds, identifying genomic regions and candidate genes associated with this economically important trait while accounting for the population structure inherent in mixed-breed samples.

## 2. Materials and Methods

**Ethics Statement.** The collection of biological samples was conducted in accordance with protocols approved by the Ethics Committee of the Research and Development Station for Bovine Arad (Statement no. 88/4 October 2019). All procedures adhered to institutional guidelines for animal care and Romanian legislation on animal welfare.

**Study Population and Sample Collection.** The study included 555 dual-purpose lactating cows comprising Romanian Simmental and Romanian Brown breeds from the Research and Development Station for Bovine Arad, of which 313 animals (271 Romanian Simmental, 42 Romanian Brown) with both high-quality genotypes and adjusted phenotypes were retained for association testing. Blood samples were collected between 2020 and 2025 through coccygeal venipuncture using EDTA-coated tubes. Samples were maintained at 4 °C immediately following collection and transferred to IFN Schönow GmbH (Bernau bei Berlin, Germany) for DNA extraction and genotyping procedures.

**Phenotype Assessment and Statistical Adjustment.** Milk samples were obtained during routine monthly recording sessions from 2020 to 2025. Protein content (expressed as a percentage) was quantified using infrared spectroscopy methodology. To determine protein content (%), milk samples were collected every 28 days by the Official Dairy Control service. The milk samples were analyzed for protein content in the laboratory of the Milk Quality Control Foundation (Cluj-Napoca, Romania) using the CombiFoss™ FT +, which integrates the MilkoScan™ FT + and Fossomatic™ FC instruments.

Phenotypic data quality control was implemented using R statistical software v4.4.0 [17] with the dplyr package v1.1.4 for data manipulation [18] and data.table v1.17.4 for efficient data processing [19]. The quality control protocol consisted of (1) elimination of zero-value protein records, (2) breed-stratified outlier identification using the interquartile range method, where values exceeding Q1 − 1.5 × IQR or Q3 + 1.5 × IQR boundaries were excluded, and (3) inclusion of only animals with a minimum of three test-day measurements. Environmental and systematic effects were adjusted using a linear mixed model using the lme4 package v1.1.37 [20] with hypothesis testing performed via lmerTest v3.1.3 [21]:

**Y_ijklmn = μ + Breed_i + DIM_j + Season_k + Year_l + Parity_m + Animal_n + e_ijklmn**

where

**Y_ijklmn** represents protein percentage;

**μ** denotes the population mean;

**Breed_i** is the fixed breed effect (Romanian Simmental or Romanian Brown);

**DIM_j** represents days in milk categories (0–100, 100–200, >200 days);

**Season_k** denotes seasonal effects (Winter, Spring, Summer, Autumn) determined using lubridate v1.9.4 [22];

**Year_l** represents the year effect;

**Parity_m** indicates parity number (1–13);

**Animal_n** represents the random animal effect;

**e_ijklmn** is the residual error.

The adjusted phenotypes were calculated as model residuals plus the population mean (3.533%). For GWAS analysis, individual animal phenotypes were derived as the mean of all adjusted test-day values for each animal, requiring a minimum of three test-day measurements. This approach yielded a single adjusted protein percentage value per animal, reducing measurement error through averaging of multiple records while maintaining the adjustment for environmental effects.

**Genotyping and Quality Control Procedures.** Genomic DNA extraction from blood samples followed standard protocols at IFN Schönow GmbH. Genotyping was conducted using the Axiom Bovine Genotyping v3 Array (Thermo Fisher Scientific, Waltham, MA, USA), which interrogates over 63,000 single-nucleotide polymorphisms distributed across the bovine genome based on the UMD 3.1.1 reference assembly [23].

From the initial cohort of 1348 dual-purpose cows with phenotypic records collected between 2020 and 2025, genotype data were available for 555 individuals (41.2% of phenotyped animals) across three genotyping batches conducted between 2020 and 2024 as funding became available. The 1348 phenotyped animals represent the complete population of Romanian Simmental and Romanian Brown cows maintained at the Research and Development Station for Bovine Arad during the study period. Affymetrix-format genotype files were converted to PLINK format using custom R scripts incorporating readxl v1.4.5 [24] and data.table v1.17.4 [19]. Quality control filtering was performed using PLINK v1.9.0 [25,26], applying the following criteria (thresholds) for quality:

1. SNP call rate exceeding 90% as recommended by McClure et al. (2018), resulting in the removal of 18,972 SNPs [27];
2. Minor allele frequency above 0.05 following Teng et al. (2023), eliminating 18,986 SNPs [28];
3. Hardy–Weinberg equilibrium *p*-value > 1 × 10^−6^ based on Zhang et al. (2016), excluding 1333 SNPs [29];
4. Elimination of 1113 duplicate SNP positions;
5. Restriction to autosomal chromosomes (BTA1-29).

Following integration of genotypic and phenotypic datasets, 313 animals (271 Romanian Simmental, 42 Romanian Brown) with both high-quality genotypes and adjusted phenotypes were retained for association testing.

**Population Structure Assessment and Association Testing.** To control for population stratification effects, principal component analysis was carried out using PLINK v1.9.0 [25] on the linkage disequilibrium-pruned dataset. LD pruning employed a 50-SNP sliding window with 5-SNP steps and an *r^2^* threshold of 0.5, yielding 33,531 independent variants. The initial ten principal components were computed, with the first five components explaining 58.7% of genetic variation and incorporated as covariates in the association model. Pedigree information was not systematically recorded for all animals in the study population, precluding formal pedigree-based relatedness estimation. Consequently, we did not employ pedigree-based mixed models that could have provided additional control for genetic relationships. Instead, population structure and cryptic relatedness were accounted for through principal component analysis (PCA) on the LD-pruned genomic data. Genome-wide association testing was conducted using PLINK’s linear regression framework [25]:

**Y = β_0_ + β_1_ × SNP + β_2_ × Breed + β_3_ × PC1 + … + β_7_ × PC5 + ε**

where Y represents the mean adjusted protein phenotype per animal derived from multiple test-day records as described above, SNP indicates allele dosage (0, 1, or 2), Breed is coded as 1 for Romanian Simmental and 2 for Romanian Brown, and PC1-PC5 represent the first five principal components. The inclusion of breed as a covariate, despite its prior inclusion in phenotype adjustment, serves to control residual population stratification not fully captured by the principal components. The genomic inflation factor (*λ*) was computed as the ratio of the observed median *χ^2^* statistic to the expected median under the *χ^2^* distribution.

**Gene Annotation and Functional Characterization.** We applied a dual-threshold approach to balance discovery power with false positive control in this exploratory GWAS. SNPs achieving *p* < 2.98 × 10^−5^ (1/33,531) were classified as significant, corresponding to one expected false positive per genome scan, adapting the ‘suggestive linkage’ concept of Lander and Kruglyak (1995) [26] to a GWAS context. Additionally, given our limited sample size (n = 313) and the exploratory nature of this GWAS in Romanian dual-purpose cattle, we report SNPs with *p* < 1 × 10^−4^ as nominal associations, representing candidate regions for validation in larger cohorts [30]. Gene annotation was performed using biomaRt v2.62.1 [31], accessing the Ensembl database (release 80, May 2015) with the UMD 3.1.1 assembly. Genes within a 100 kb window of significant SNPs [30,32] were identified using custom R scripts implementing httr v1.4.7 [33] and jsonlite v2.0.0 [34].

**Data Visualization and Statistical Computing.** Manhattan and quantile-quantile plots were generated using ggplot2 v3.5.2 [35] with publication-quality formatting through cowplot v1.1.3 [36] and multi-panel arrangements via grid Extra v2.3 [37]. Result tables were exported using writexl v1.5.4 [38]. All statistical computations were performed in R v4.4.0 [17]. Quality control procedures and association analyses were executed using PLINK v1.9.0-beta7.11 [25,39].

**Generative artificial intelligence** (Claude AI, Anthropic; Claude Opus 4.1 model) was used in the preparation of this manuscript for the following purposes: (1) prototyping and debugging R code for data manipulation, quality control procedures, and GWAS analysis implementation; (2) troubleshooting errors in statistical scripts and optimizing code efficiency for principal component analysis and linear mixed model implementation; (3) assistance in proper syntax and parameter specification for R packages including PLINK integration, lme4 model construction, and data visualization using ggplot2. The AI system was not given access to raw data and did not perform any independent statistical analysis or interpretation. The authors determined all research findings and their biological significance exclusively.

## 3. Results

### 3.1. Phenotype Distribution and Population Characteristics

The final dataset comprised 313 dual-purpose cows, including 271 Romanian Simmental (86.6%) and 42 Romanian Brown (13.4%) animals with both high-quality genotypes and adjusted protein phenotypes. Following adjustment for other effects (breed, days in milk, season, year, and parity), the mean milk protein content was 3.533 ± 0.068% (range: 3.125–3.923%). The adjusted protein values were normally distributed (skewness = −0.12, kurtosis = 2.87) and did not differ significantly between breeds (Romanian Simmental: 3.542 ± 0.051%; Romanian Brown: 3.488 ± 0.112%; Welch’s *t*-test, *p* = 0.082).

### 3.2. Population Structure Analysis

**Figure 1:**
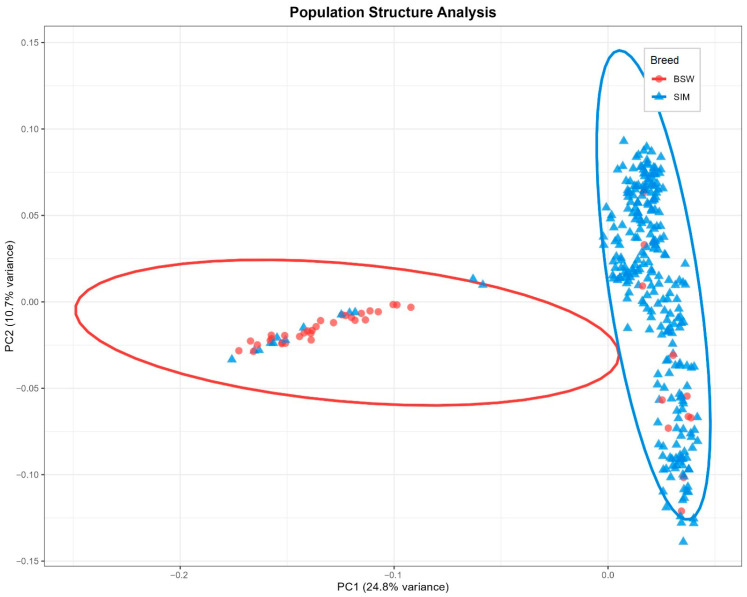
Population structure analysis of 313 Romanian dual-purpose cattle. **Legend:** Principal component analysis showing clear separation between Romanian Simmental (blue, n = 271) and Romanian Brown (red, n = 42) breeds along PC1 (24.8% variance) and PC2 (10.7% variance).

**Figure 2:**
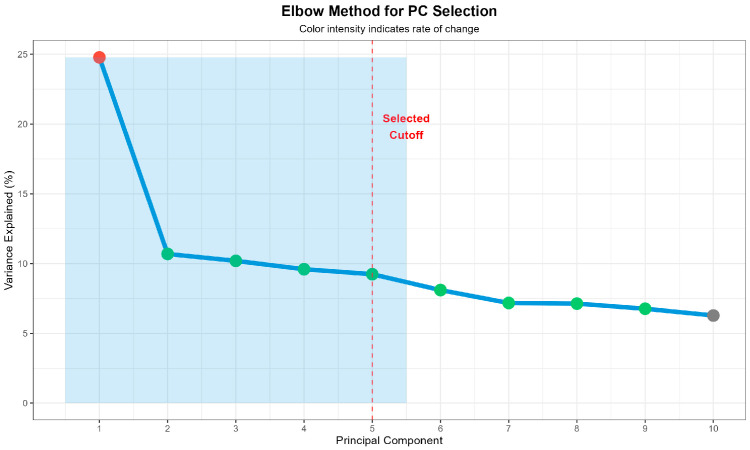
Elbow method via scree plot for principal component selection in the GWAS analysis. **Legend:** Variance explained by the first 10 principal components is shown, with PC1 highlighted in red, PC2–PC9 in green, and PC10 in grey. The first five components (PC1–PC5, blue shaded area) were selected as covariates, based on an elbow point identified at PC5 (red dashed line). These five components cumulatively explain 58.7% of genetic variation in the study population.

Principal component analysis revealed distinct population stratification corresponding to breed structure (Figure 1). The first principal component (PC1) explained 24.8% of the genetic variation and effectively separated Romanian Simmental from Romanian Brown cattle populations. PC2 accounted for 10.7% of the variation, with within-breed substructure evident particularly in the Simmental population. The scree plot analysis indicated that the first five principal components cumulatively explained 58.7% of the total genetic variation, with a clear elbow point after PC5, supporting their inclusion as covariates in the association model (Figure 2).

### 3.3. Genome-Wide Association Analysis

**Figure 3:**
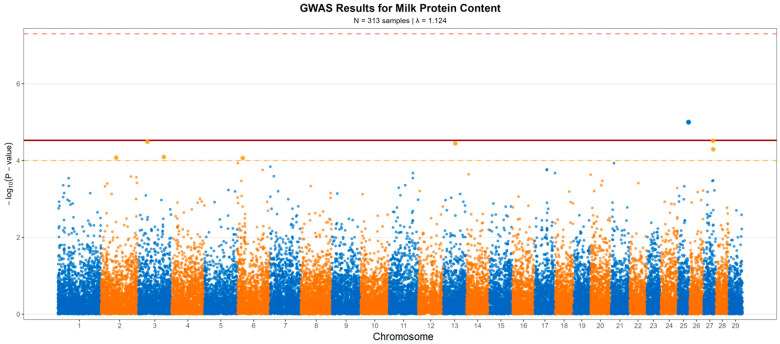
Manhattan plot of genome-wide association results for milk protein content. **Legend:** Distribution of −log_10_ (*p*-values) for 33,531 SNPs across 29 bovine autosomes. The upper red dashed line indicates the genome-wide significance threshold (*p* = 5 × 10^−8^), the lower orange dashed line indicates the nominal significance threshold (*p* = 1 × 10^−4^), and the continuous red line indicates the significance threshold (*p* < 2.98 × 10^−5^).

**Figure 4:**
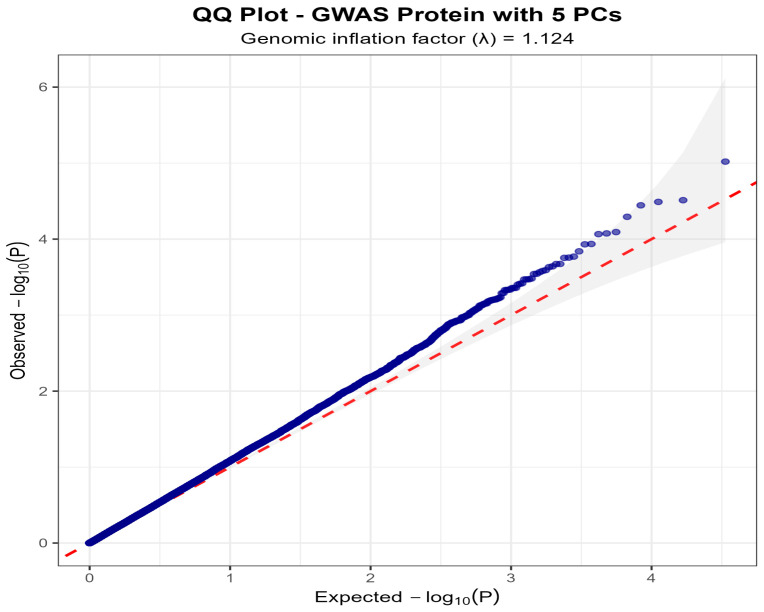
Quantile-quantile plot for the GWAS analysis. **Legend:** Q plot showing observed versus expected −log_10_ (*p*-values) with genomic inflation factor *λ* = 1.124. Blue dots represent the observed *p*-values from the association analysis. The red dashed line indicates the expected distribution under the null hypothesis of no association (y = x line). The gray shaded area represents the 95% confidence interval for the expected distribution. Deviation of points from the diagonal line, particularly in the tail of the distribution, suggests the presence of true associations.

After quality control and LD pruning, 33,531 SNPs were tested for association with milk protein content. The genomic inflation factor (*λ* = 1.124) indicated moderate population stratification despite the mixed-breed sample (Figure 2 and Figure 3). The quantile-quantile plot demonstrated deviation from the expected distribution primarily in the tail region, consistent with true associations rather than systematic inflation (Figure 4). None of the SNPs reached the conventional genome-wide significance threshold of *p* < 5 × 10^−8^. However, eight SNPs exceeded the nominal significance threshold (*p* < 1 × 10^−4^), distributed across six chromosomes (Figure 3, Table 1). The strongest association was observed for SNP AX-115120431 on chromosome 25 (*p* < 1 × 10^−5^, β = −0.0301%, MAF = 0.299), located within the 5′ region of the *C7orf61* gene, which achieved significance under (*p* = 9.56 × 10^−6^ < 2.98 × 10^−5^).

### 3.4. Chromosomal Distribution and Functional Annotation

The eight SNPs (one significant, seven nominal) were distributed across chromosomes 2, 3, 6, 13, 25, and 27, with chromosomes 3 and 27 harboring two independent signals (Table 1). Six SNPs mapped to protein-coding genes, while one was intergenic and one had an ambiguous allele assignment.

Three SNPs were located within the gene: AX-115120431 in *C7orf61*, AX-185117724 in ZMAT4, and AX-117081548 in SLC1A7. The remaining SNPs were located at varying distances from their nearest genes, ranging from 2.7 kb (*AHCYL1*) to 96.6 kb (ZMAT4 distant signal).

Effect sizes ranged from −0.0301% to 0.0292% per allele copy. Four SNPs (AX-115120431, AX-106725208, AX-117081548, and AX-106749405) showed negative effects (associated with decreased protein content), while four showed positive effects. Minor allele frequencies ranged from 0.085 (AX-185117724) to 0.498 (AX-185118865), indicating that both rare and common variants contribute to the genetic architecture of milk protein content in this population.

## 4. Discussion

This study represents a genome-wide association analysis of milk protein content in Romanian dual-purpose cattle, analyzing 313 dual-purpose animals from Romanian Simmental and Romanian Brown populations. The identification of eight SNPs distributed across six chromosomes provides initial insights into the genetic architecture of milk protein synthesis in these locally adapted breeds, though the relatively modest sample size limited the power to detect genome-wide significant associations in our study.

**Population Structure.** The genomic inflation factor of *λ* = 1.124 observed in our study falls within the moderate range (1.1–1.2) as classified by Panagiotou et al. (2016) and Doherty et al. (2018), where such values indicate moderate influence on statistical power in meta-analyses of genome-wide association studies [17,40]. Control of population structure through principal component analysis determined that the first five components explain 58.7% of genetic variation.

The PCA plot (Figure 1) reveals several individuals clustering with the opposite breed group, an observation that warrants biological interpretation. This can be explained by historical gene flow: breeding programs have periodically introduced Simmental genetics into Brown populations and vice versa to improve production traits while maintaining breed standards, potentially creating admixed individuals registered within one breed but carrying substantial genomic ancestry from the other.

The genomic inflation factor of *λ* = 1.124 represents a clear limitation of our study, indicating moderate residual population stratification despite correction with principal components. This residual confounding may have inflated test statistics, potentially contributing to the identification of biologically implausible associations such as *C7orf61* (testis-specific) and *SLC1A7* (retina-specific). The inflation factor suggests that some of our nominal associations may represent false positives arising from incomplete correction of cryptic relatedness or population substructure rather than true biological effects. Future studies should consider alternative approaches, such as linear mixed models with genomic relationship matrices, to better control for this residual stratification.

**Candidate Genes and Biological Relevance.** The strongest association signal (*p* = 9.56 × 10^−6^) was mapped to the *C7orf61* gene on chromosome 25. No mammary or lactation functions have been documented for *C7orf61*. This gene has been characterized exclusively as a testis-specific factor with roles restricted to spermatogenesis and gamete fusion in mouse and human models [41]. The biological implausibility of this association with milk protein content, combined with our LD pruning approach, suggests this signal likely represents either a false positive arising from limited sample size or linkage with an unmeasured causal variant in a neighboring locus not captured by our genotyping array.

*KLF6* (Krüppel-like factor 6) on chromosome 13 is a promising candidate gene. Research in Chinese Holstein cattle has established significant correlations between twelve *KLF6* polymorphisms and multiple production traits, including milk yield, fat content, and protein parameters, with individual variants accounting for phenotypic variance ranging from negligible values to approximately 2.13% [42]. As a transcription factor with known roles in cellular differentiation and metabolic regulation, *KLF6* represents a biologically plausible candidate for influencing milk composition traits, though direct functional validation of its role in mammary tissue metabolism remains to be performed.

*AHCYL1* (S-adenosylhomocysteine hydrolase-like 1), positioned 2.7 kb from our associated SNP on chromosome 3, functions as a regulator of intracellular calcium dynamics through its interaction with inositol 1,4,5-trisphosphate receptors in chickens [43]. Although direct evidence linking *AHCYL1* to bovine lactation traits remains absent, its responsiveness to estrogen signaling via ERK1/2 MAPK pathways and fundamental role in calcium homeostasis suggest potential involvement in mammary epithelial cell function. The calcium-sensing receptor has been demonstrated to regulate both parathyroid hormone-related protein synthesis and transcellular calcium transport in mammary epithelial cells [44], establishing calcium signaling as critical for milk protein secretion and making *AHCYL1* a biologically relevant candidate requiring further investigation.

The *SLC1A7* gene encodes excitatory amino acid transporter 5 (EAAT5), which exhibits highly restricted expression in retinal tissue, where it functions primarily as a glutamate-gated chloride channel in humans rather than a classical amino acid transporter [45]. Direct examination of *SLC1A7* in mammary tissue has not been reported. The restricted retinal expression pattern of *SLC1A7* and the absence of mammary-specific characterization suggest either a false positive caused by limited sample size or linkage with an unmeasured causal variant in a neighboring locus not captured by our genotyping array.

*ZMAT4*, identified through SNPs on chromosome 27, encodes a zinc finger matrin-type protein that has been involved in transcriptional regulation, but lacks any documented characterization in mammary biology. The Human Protein Atlas database indicates tissue-specific enrichment limited to brain, retina, and thyroid gland, with no detectable mammary tissue expression [46]. Despite the absence of *ZMAT4*-specific lactation studies, research on other zinc finger proteins highlights their potential importance: the zinc finger homeodomain factor Zfhx3 maintains prolactin receptor expression and *STAT5* signaling activity, with genetic ablation causing complete lactation failure through disrupted lactogenesis [47]. This precedent suggests zinc finger proteins warrant systematic investigation in milk production contexts, though direct evidence for *ZMAT4* involvement remains absent.

*DKK2*, located near our associated SNP on chromosome 6, functions as an antagonist of Wnt signaling pathways. Research has demonstrated that *DKK2* inhibits canonical Wnt/β-catenin signaling through suppression of β-catenin protein activity in humans [48]. While Wnt signaling plays essential roles in mammary gland development, comprehensive reviews examining established quantitative trait loci for milk production in cattle do not include *DKK2* among recognized candidate genes [49]. The absence of functional studies linking *DKK2* to milk protein synthesis during lactation necessitates comprehensive validation through expression profiling across lactation stages and functional studies in bovine mammary epithelial cells before biological relevance can be established.

**Comparison with Established QTL Regions.** Notably absent from our results are the major QTL consistently identified in other cattle populations. The casein gene cluster on BTA6, containing CSN1S1, CSN1S2, CSN2, and CSN3, shows the strongest effects on milk protein composition across breeds [6,7]. Similarly, the DGAT1 gene on BTA14, explaining substantial variance in milk composition traits [8], showed no significant associations in our analysis. The PAEP gene on BTA11, encoding β-lactoglobulin, which shows heritability of 0.80 in Dutch Holstein-Friesians [5], also failed to reach significance.

This discrepancy may be attributed to several factors: (1) different allele frequencies in Romanian breeds compared to intensively selected dairy populations, (2) insufficient statistical power to detect variants with moderate effects, (3) potential breed-specific genetic architecture for milk protein content, or (4) linkage disequilibrium patterns unique to Romanian cattle affecting marker-QTL associations. The high genetic diversity observed in Romanian breeds [10], combined with less intensive selection compared to mainstream dairy breeds, may contribute to different genetic architectures.

While our study included only Romanian Simmental and Brown cattle types due to sample availability, the methodology established here provides a framework for future genomic studies in Romanian dual-purpose cattle when sufficient samples become available. The identification of potentially breed-specific variants influencing milk protein content supports maintaining genetic diversity through conservation programs rather than replacing local breeds with mainstream dairy cattle.

**Limitations and Future Directions.** Several limitations should be considered when interpreting our results. The mixed-breed sample, while increasing overall numbers, may have reduced power to detect breed-specific effects. The relatively small sample size limited our ability to detect variants with small effects that collectively contribute to trait variation. Additionally, the use of a commercial SNP array designed primarily for *Bos taurus* breeds may have missed rare variants specific to Romanian populations. The genomic inflation factor (*λ* = 1.124), while moderate, suggests residual population stratification that may have influenced our results despite correction with principal components.

Future studies should prioritize (1) increasing sample sizes through collaborative efforts among Romanian research institutions, (2) implementing imputation strategies to increase marker density, (3) conducting functional validation of candidate genes through expression studies in mammary tissue, and (4) investigating gene-by-environment interactions specific to Romanian production systems. Whole-genome sequencing of key individuals could identify breed-specific variants not captured by commercial arrays.

## 5. Conclusions

This GWAS of milk protein content in Romanian dual-purpose cattle identifies several genomic regions potentially influencing this economically important trait. While the modest sample size precluded detection of genome-wide significant associations, the nominal associations near *KLF6* and *AHCYL1*, while not reaching stringent significance thresholds, deserve validation in larger cohorts given their biological plausibility. The absence of associations at major QTL identified in other breeds suggests potential breed-specific genetic architecture in Romanian cattle, emphasizing the importance of conducting population-specific genomic studies. Our results provide a foundation for developing genomic selection programs adapted to Romanian dual-purpose cattle while contributing to global understanding of the genetic basis of milk protein synthesis. Continued research with larger sample sizes and functional validation will be essential to fully elucidate the genetic architecture of milk production traits in these valuable local genetic resources.

## Figures and Tables

**Table 1:** SNPs (*p* < 1 × 10^−4^) associated with milk protein content in Romanian dual-purpose cattle.

Rank	SNP ID	Chr	Position (bp)	EA/OA	MAF	β (SE)	*p*-Value	Gene	Location
1	AX-115120431	25	36,625,127	A/C	0.299	−0.0301 (0.0067)	9.56 × 10^−6^	*C7orf61*	Within gene
2	AX-106740068	27	35,144,687	G/A	0.264	0.0199 (0.0047)	3.08 × 10^−5^	*ZMAT4*	96.6 kb
3	AX-106725208	3	33,559,007	G/A	0.296	−0.0190 (0.0045)	3.24 × 10^−5^	*AHCYL1*	2.7 kb
4	AX-106756277	13	44,876,436	A/G	0.178	0.0257 (0.0061)	3.59 × 10^−5^	*KLF6*	68.6 kb
5	AX-185117724	27	35,382,031	C/T	0.085	0.0292 (0.0071)	5.09 × 10^−5^	*ZMAT4*	Within gene
6	AX-117081548	3	93,682,810	G/T	0.315	−0.0193 (0.0048)	8.08 × 10^−5^	*SLC1A7*	Within gene
7	AX-185118865	2	55,947,021	C/C	0.498	0.0173 (0.0043)	8.41 × 10^−5^	Intergenic	-
8	AX-106749405	6	19,518,197	C/T	0.411	−0.0166 (0.0042)	8.60 × 10^−5^	*DKK2*	7.0 kb

Legend: SNP ID = identifier assigned to a specific Single Nucleotide Polymorphism (SNP); Chr = chromosomes; bp = base pairs; EA = effect allele; OA = other allele; MAF = Minor allele frequencies; β = effect size in percentage points per copy of the effect allele; and SE = standard error.

## Data Availability

The data that support the findings of this study are not publicly available due to a confidentiality agreement with the Research and Development Station for Bovine Arad that restricts the sharing of individual animal genotype and phenotype data. Researchers interested in collaboration or additional information may contact the corresponding author, who will facilitate communication with the data custodians, subject to appropriate data sharing agreements.

## Abbreviations

The following abbreviations are used in this manuscript:

AHCYL1	S-Adenosylhomocysteine Hydrolase-Like 1
BTA	Bos Taurus Autosome
CNCS-UEFISCDI	National Council for Scientific Research-Executive Agency for Higher Education, Research, Development, and Innovation Funding
CSN	Casein (CSN1S1, CSN1S2, CSN2, CSN3-casein gene variants)
DGAT1	Diacylglycerol O-Acyltransferase 1
DIM	Days In Milk
DKK2	Dickkopf-related protein 2
DNA	Deoxyribonucleic Acid
EA	Effect Allele
EAAT5	Excitatory Amino Acid Transporter 5
EDTA	Ethylenediaminetetraacetic Acid
ERK1/2	Extracellular signal-Regulated Kinase 1/2
GWAS	Genome-Wide Association Studies
IP3	Inositol 1,4,5-trisphosphate
IQR	Interquartile Range
KLF6	Krüppel-Like Factor 6
LD	Linkage Disequilibrium
LIC	Livestock Improvement Corporation
MAF	Minor Allele Frequency
MAPK	Mitogen-Activated Protein Kinase
NZD	New Zealand Dollar
OA	Other Allele
PAEP	Progestagen-Associated Endometrial Protein (β-lactoglobulin)
PC	Principal Component
PCA	Principal Component Analysis
PNCDI	National Plan for Research, Development, and Innovation
PTHrP	Parathyroid Hormone-related Protein
QQ	Quantile-Quantile
QTL	Quantitative Trait Loci
SE	Standard Error
SLC1A7	Solute Carrier Family 1 Member 7
SNP	Single Nucleotide Polymorphism
STAT5	Signal Transducer and Activator of Transcription 5
UMD	University of Maryland (bovine genome assembly)
ZMAT4	Zinc finger Matrin-Type 4

## References

[1] LIC (2025). Economic Values in the New Zealand Dairy Industry. Livestock Improvement Corporation. https://www.lic.co.nz/about/supporting-our-industry/animal-evaluation/about-animal-evaluation/.

[2] Huțu I. (2015). Considerations on Lactation in Cattle under Romanian Formal Recording. Lucr. Științifice Med. Vet. Timișoara.

[3] Lu X., Arbab A.A.I., Abdalla I.M., Liu D., Zhang Z., Xu T., Su G., Yang Z. (2021). Genetic Parameter Estimation and Genome-Wide Association Study-Based Loci Identification of Milk-Related Traits in Chinese Holstein. Front. Genet..

[4] Buitenhuis B., Poulsen N.A., Gebreyesus G., Larsen L.B. (2016). Estimation of Genetic Parameters and Detection of Chromosomal Regions Affecting the Major Milk Proteins and Their Post-Translational Modifications in Danish Holstein and Danish Jersey Cattle. BMC Genet..

[5] Schopen G.C.B., Heck J.M.L., Bovenhuis H., Visker M.H.P.W., van Valenberg H.J.F., van Arendonk J.A.M. (2009). Genetic Parameters for Major Milk Proteins in Dutch Holstein-Friesians. J. Dairy Sci..

[6] van den Berg I., Xiang R., Jenko J., Pausch H., Boussaha M., Schrooten C., Tribout T., Gjuvsland A.B., Boichard D., Nordbø Ø. (2020). Meta-Analysis for Milk Fat and Protein Percentage Using Imputed Sequence Variant Genotypes in 94,321 Cattle from Eight Cattle Breeds. Genet. Sel. Evol..

[7] Pegolo S., Mach N., Ramayo-Caldas Y., Schiavon S., Bittante G., Cecchinato A. (2018). Integration of GWAS, Pathway and Network Analyses Reveals Novel Mechanistic Insights into the Synthesis of Milk Proteins in Dairy Cows. Sci. Rep..

[8] Jiang J., Ma L., Prakapenka D., VanRaden P.M., Cole J.B., Da Y. (2019). A Large-Scale Genome-Wide Association Study in U.S. Holstein Cattle. Front. Genet..

[9] Ilie D.E., Mizeranschi A.E., Mihali C.V., Neamț R.I., Goilean G.V., Georgescu O.I., Zaharie D., Carabaș M., Huțu I. (2021). Genome-Wide Association Studies for Milk Somatic Cell Score in Romanian Dairy Cattle. Genes.

[10] Ilie D.E., Cean A., Cziszter L.T., Gavojdian D., Ivan A., Kusza S. (2015). Microsatellite and Mitochondrial DNA Study of Native Eastern European Cattle Populations: The Case of the Romanian Grey. PLoS ONE.

[11] Davidescu M.A., Simeanu D., Gorgan D.L., Ciorpac M., Creangă S. (2022). Analysis of Phylogeny and Genetic Diversity of Endangered Romanian Grey Steppe Cattle Breed, a Reservoir of Valuable Genes to Preserve Biodiversity. Agriculture.

[12] Cziszter L.T., Gavojdian D., Neamț R.I., Neciu F.C., Saplacan S.I., Ilie D.E. (2017). Comparative Study on Production, Reproduction and Functional Traits Between Fleckvieh and Braunvieh Cattle. Asian-Australas. J. Anim. Sci..

[13] Grădinaru A.C., Petrescu-Mag I.V., Oroian F.C., Balint C., Oltean I. (2018). Milk Protein Polymorphism Characterization—A Modern Tool for Sustainable Conservation of Endangered Romanian Cattle Breeds in the Context of Traditional Breeding. Sustainability.

[14] Neamț R.I., Saplacan G., Acatincai S., Cziszter L.T., Gavojdian D., Ilie D.E. (2017). The Influence of CSN3 and LGB Polymorphisms on Milk Production and Chemical Composition in Romanian Simmental Cattle. Acta Biochim. Pol..

[15] Ilie D.E., Mizeranschi A.E., Mihali C.V., Neamț R.I., Cziszter L.T., Carabaș M., Grădinaru A.C. (2023). Polymorphism of the Prolactin (PRL) Gene and Its Effect on Milk Production Traits in Romanian Cattle Breeds. Vet. Sci..

[16] Panagiotou O.A., Evangelou E., Ioannidis J.P. (2013). Genome-wide Significant Associations for Variants with Minor Allele Frequency of 5% or Less—An Overview: A HuGE Review. Annu. Rev. Genom. Hum. Genet..

[17] R Core Team (2024). R: A Language and Environment for Statistical Computing.

[18] Wickham H., François R., Henry L., Müller K., Vaughan D. (2023). dplyr: A Grammar of Data Manipulation.

[19] Barrett T., Dowle M., Srinivasan A., Gorecki J., Chirico M., Hocking T., Schwendinger B. (2023). data.table: Extension of ‘data.frame’.

[20] Bates D., Mächler M., Bolker B., Walker S. (2015). Fitting Linear Mixed-Effects Models Using lme4. J. Stat. Softw..

[21] Kuznetsova A., Brockhoff P.B., Christensen R.H.B. (2017). lmerTest Package: Tests in Linear Mixed Effects Models. J. Stat. Softw..

[22] Grolemund G., Wickham H. (2011). Dates and Times Made Easy with lubridate. J. Stat. Softw..

[23] Zi-min A.V., Delcher A.L., Florea L., Kelley D.R., Schatz M.C., Puiu D., Hanrahan F., Pertea G., Van Tassell C.P., Sonstegard T.S. (2009). A Whole-Genome Assembly of the Domestic Cow, *Bos taurus*. Genome Biol..

[24] Wickham H., Bryan J. (2025). readxl: Read Excel Files.

[25] Purcell S., Neale B., Todd-Brown K., Thomas L., Ferreira M.A.R., Bender D., Maller J., Sklar P., de Bakker P.I.W., Daly M.J. (2007). PLINK: A Tool Set for Whole-Genome Association and Population-Based Linkage Analyses. Am. J. Hum. Genet..

[26] Lander E.S., Kruglyak L. (1995). Genetic Dissection of Complex Traits: Guidelines for Interpreting and Reporting Linkage Results. Nat. Genet..

[27] McClure M.C., McCarthy J., Flynn P., McClure J.C., Dair E., O’Connell D.K., Kearney J.F. (2018). SNP Data Quality Control in a National Beef and Dairy Cattle System and Highly Accurate SNP-Based Parentage Verification and Identification. Front. Genet..

[28] Teng J., Wang D., Zhao C., Zhang X., Chen Z., Liu J., Sun D., Tang H., Wang W., Li J. (2023). Longitudinal Genome-Wide Association Studies of Milk Production Traits in Holstein Cattle Using Whole-Genome Sequence Data Imputed from Medium-Density Chip Data. J. Dairy Sci..

[29] Zhang F., Wang Y., Mukiibi R., Chen L., Vinsky M., Plastow G., Basarab J., Stothard P., Li C. (2016). Pathway-Based Genome-Wide Association Studies for Two Meat Production Traits in Simmental Cattle. Sci. Rep..

[30] Coppa L., Khanal P., Pant S., Nakano A.H., Austin K.J., Cammack K.M., Lee J., Murdoch B.M. (2023). Genome-Wide Association Study for Carcass Weight in Pasture-Finished Beef Cattle in Hawai’i. Front. Genet..

[31] Durinck S., Spellman P.T., Birney E., Huber W. (2009). Mapping Identifiers for the Integration of Genomic Datasets with the R/Bioconductor Package biomaRt. Nat. Protoc..

[32] Kim M.-S., Ko S.-R., Le V.T., Jee M.-G., Jung Y.J., Kang K.-K., Cho Y.-G. (2022). Development of SNP Markers from GWAS for Selecting Seed Coat and Aleurone Layers in Brown Rice (*Oryza sativa* L.). Genes.

[33] Wickham H. (2023). httr: Tools for Working with URLs and HTTP. R Package Version 1.4.7..

[34] Ooms J. (2014). The jsonlite Package: A Practical and Consistent Mapping Between JSON Data and R Objects. arXiv.

[35] Wickham H. (2016). ggplot2: Elegant Graphics for Data Analysis.

[36] Wilke C.O. (2024). cowplot: Streamlined Plot Theme and Plot Annotations for ‘ggplot2’.

[37] Auguie B., Antonov A. (2017). gridExtra: Miscellaneous Functions for “Grid” Graphics.

[38] Ooms J. (2025). writexl: Export Data Frames to Excel ‘xlsx’ Format.

[39] Chang C.C., Chow C.C., Tellier L.C., Vattikuti S., Purcell S.M., Lee J.J. (2015). Second-Generation PLINK: Rising to the Challenge of Larger and Richer Datasets. GigaScience.

[40] Doherty A., Smith-Byrne K., Ferreira T., Holmes M.V., Holmes C., Pulit S.L., Lindgren C.M. (2018). GWAS Identifies 14 Loci for Device-Measured Physical Activity and Sleep Duration. Nat. Commun..

[41] Wu H., Chen Y., Zhou Q., Wang R., Xia B., Ma D., Luo K., Liu Q. (2024). Testis-Specific Gene C7orf61 Is Involved in Mouse Sperm-Egg Fusion. Urol. J..

[42] Liu Y., Han B., Zheng W., Peng F., Lin Y., Ren J., Cao L., Shen Y., Zhao C., Li J. (2023). Identification of Genetic Associations and Functional SNPs of Bovine KLF6 Gene on Milk Production Traits in Chinese Holstein. BMC Genom. Data.

[43] Jeong W., Kim J., Ahn S.E., Lee S.I., Bazer F.W., Han J.Y., Song G. (2012). AHCYL1 Is Mediated by Estrogen-Induced ERK1/2 MAPK Cell Signaling and MicroRNA Regulation to Affect Functional Aspects of the Avian Oviduct. PLoS ONE.

[44] Van Houten J.N., Wysolmerski J.J. (2004). The Calcium-Sensing Receptor Regulates Mammary Gland Parathyroid Hormone-Related Protein Production and Calcium Transport. J. Clin. Investig..

[45] Arriza J.L., Eliasof S., Kavanaugh M.P., Amara S.G. (1997). Excitatory Amino Acid Transporter 5, a Retinal Glutamate Transporter Coupled to a Chloride Conductance. Proc. Natl. Acad. Sci. USA.

[46] Uhlén M., Fagerberg L., Hallström B.M., Lindskog C., Oksvold P., Mardinoglu A., Sivertsson Å., Kampf C., Sjöstedt E., Asplund A. (2015). Tissue-Based Map of the Human Proteome. Science.

[47] Zhao D., Liu S., Wang M., Zhai W., Xu Z., Wang C., Li X., Liu J., Li F. (2016). Zinc Finger Homeodomain Factor Zfhx3 Is Essential for Mammary Lactogenic Differentiation by Maintaining Prolactin Signaling Activity. J. Biol. Chem..

[48] Mu J., Hui T., Shao B., Li L., Du Z., Lu L., Ye L., Li S., Li Q., Xiao Q. (2017). Dickkopf-Related Protein 2 Induces G0/G1 Arrest and Apoptosis Through Suppressing Wnt/β-Catenin Signaling and Is Frequently Methylated in Breast Cancer. Oncotarget.

[49] Lopdell T.J. (2023). Using QTL to Identify Genes and Pathways Underlying the Regulation and Production of Milk Components in Cattle. Animals.

